# Supplementary material for: Mitochondrial mechanics nucleates axonal jamming and swelling
Source: bioRxiv. 2026 Apr 25:2026.04.23.720276. Preprint. [Version 1] doi: 10.64898/2026.04.23.720276 (PMC13131547; doi:10.64898/2026.04.23.720276)
Supplement: Supplement 9 [file NIHPP2026.04.23.720276v1-supplement-9.pdf]

bioRxiv preprint doi: https://doi.org/10.64898/2026.04.23.720276; this version posted April 25, 2026. The copyright holder for this preprint (which was not certified by peer review) is the author/funder, who has granted bioRxiv a license to display the preprint in perpetuity. It is made available under aCC-BY 4.0 International license.

10 Supplementary Material

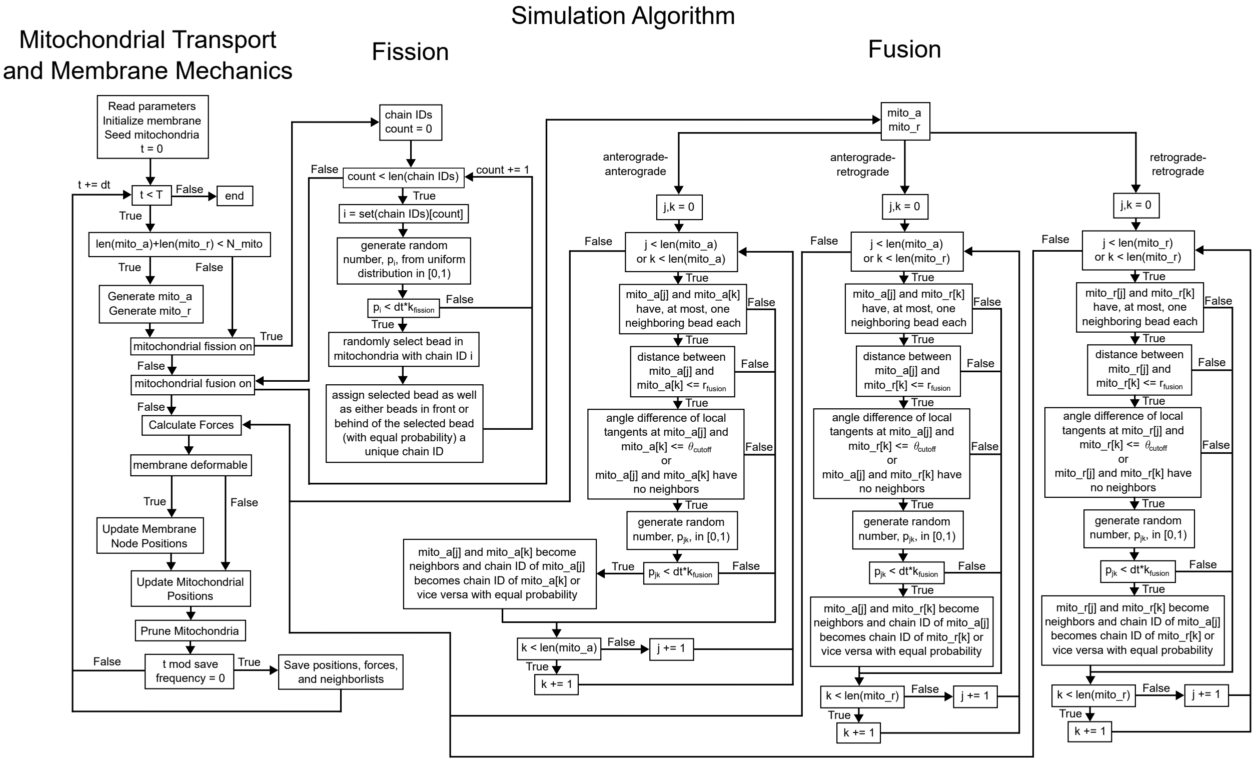

Figure S1: Graphical Algorithm of Mitochondrial transport in axon - including mitochondrial transport and membrane mechanics (left), mitochondrial fission (middle), and mitochondrial fusion (right).

bioRxiv preprint doi: https://doi.org/10.64898/2026.04.23.720276; this version posted April 25, 2026. The copyright holder for this preprint (which was not certified by peer review) is the author/funder, who has granted bioRxiv a license to display the preprint in perpetuity. It is made available under aCC-BY 4.0 International license.

Table S1: Figure Parameter Variations

Figure	Varied Parameter(s)	Values
1	-	-
2	Density (ϕ) [1]	$0.3\phi_{\text{max}}, 0.6\phi_{\text{max}}, 0.9\phi_{\text{max}}$
3	Mitochondrial bending rigidity $(k_{\text{b,mito}})$ [Nm^2]<br>Density (ϕ) [1]	$10^{-21}, 10^{-20}, 10^{-19}$<br>$0.3\phi_{\text{max}}, 0.6\phi_{\text{max}}, 0.9\phi_{\text{max}}$
4	Number of beads per mitochondria (N_{chain}) [#]<br>Density (ϕ) [1]	1, 2, 3, 4, 5<br>$0.3\phi_{\text{max}}, 0.6\phi_{\text{max}}, 0.9\phi_{\text{max}}$
5	Fusion rate (k_{fusion}) [s^{-1}]<br>Fission rate (k_{fission}) [s^{-1}]	0, 0.01, 0.1, 1.0, 10, 100<br>0, 0.01, 0.1, 1.0, 10, 100
6	Fission rate (k_{fission}) [s^{-1}]<br>Number of beads per mitochondria (N_{chain}) [#]	0, 0.1, 1, 10<br>1, 5

bioRxiv preprint doi: https://doi.org/10.64898/2026.04.23.720276; this version posted April 25, 2026. The copyright holder for this preprint (which was not certified by peer review) is the author/funder, who has granted bioRxiv a license to display the preprint in perpetuity. It is made available under aCC-BY 4.0 International license.

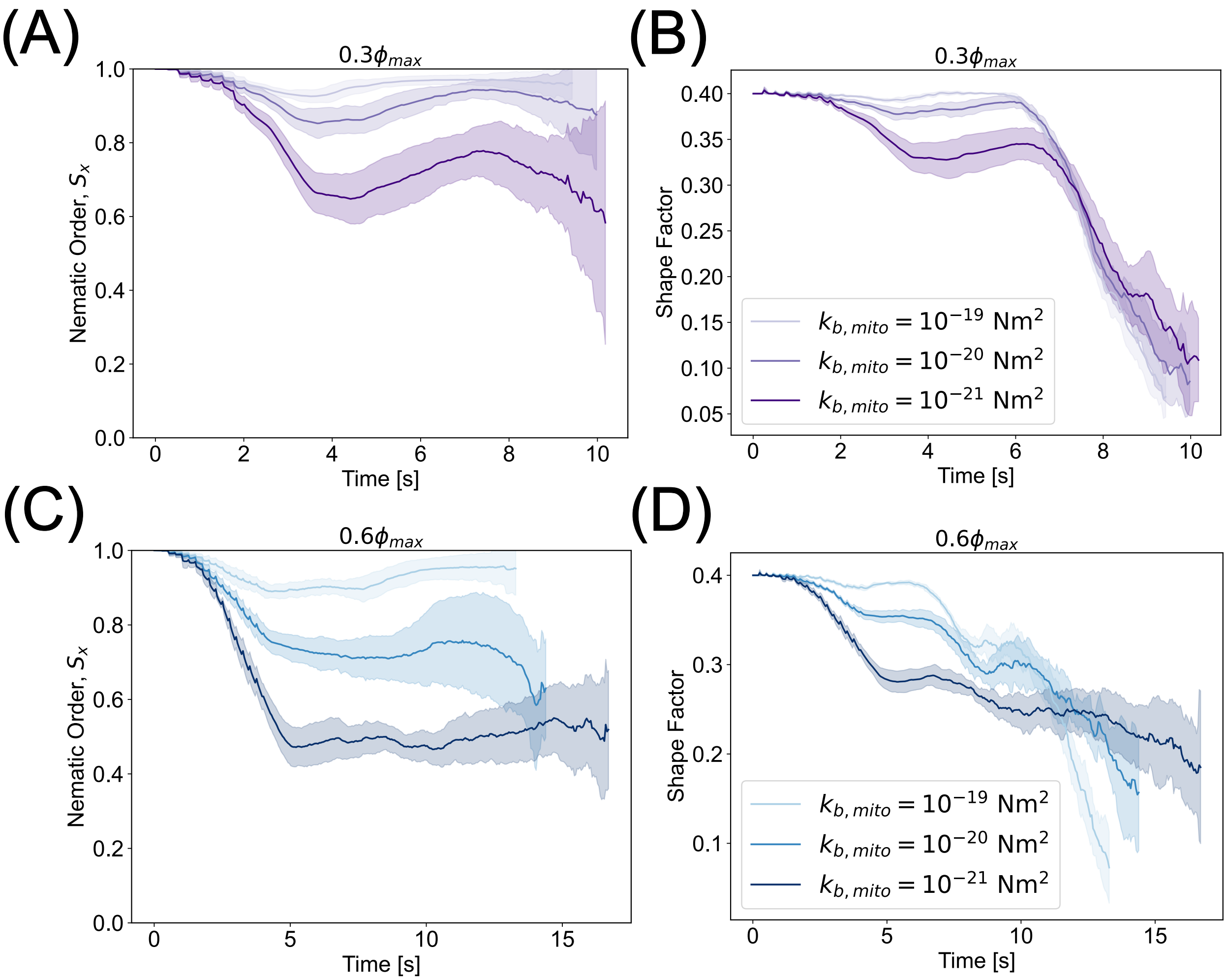

Figure S2: **Nematic and shape factor analysis of intermediate and sparse densities for varying bending stiffness.** (A,B) Nematic order and shape factor of lowest densities, $\phi = 0.3\phi_{\max}$, shows 10^{-19} Nm^2 and 10^{-20} Nm^2 bending rigidities recover initial morphologies, while 10^{-21} Nm^2 only partially recovers. (C-D) Intermediate densities exhibit very similar behavior to their high density counterparts described in the main text. Curves and shaded regions represent the mean and standard deviation, respectively, of ten simulations per parameter set.

bioRxiv preprint doi: https://doi.org/10.64898/2026.04.23.720276; this version posted April 25, 2026. The copyright holder for this preprint (which was not certified by peer review) is the author/funder, who has granted bioRxiv a license to display the preprint in perpetuity. It is made available under aCC-BY 4.0 International license.

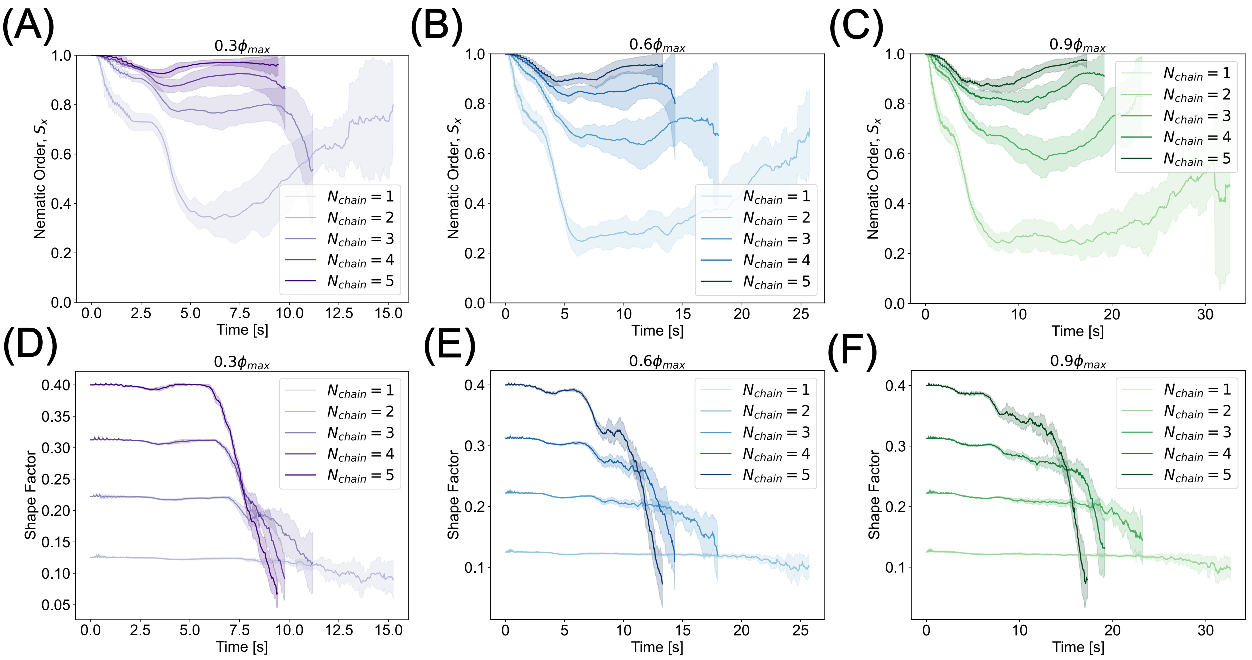

Figure S3: **Nematic and shape factor analysis of intermediate and sparse densities for varying mitochondrial aspect ratio.** (A-C) Nematic order time courses of increasing density for morphologies from granular ($N_{\text{chain}} = 1$) to elongated ($N_{\text{chain}} = 5$). Elongated mitochondria retain nematic order and recover unity while shorter morphologies show a reduced orientational order that does not recover. (D-F) Corresponding shape factors. Elongated mitochondria have a higher shape factor than shorter counterparts. Shape factor decays near the end of the trajectory as beads are exiting the simulation axon. Curves and shaded regions represent the mean and standard deviation, respectively, of ten simulations per parameter set.
